# Severe hypertriglyceridemia due to two novel loss-of-function lipoprotein lipase gene mutations (C310R/E396V) in a Chinese family associated with recurrent acute pancreatitis

**DOI:** 10.18632/oncotarget.17762

**Published:** 2017-05-10

**Authors:** Yu Lun, Xiaofang Sun, Ping Wang, Jingwei Chi, Xu Hou, Yangang Wang

**Affiliations:** ^1^ Department of Endocrinology and Metabolic Diseases, The Affiliated Hospital of Qingdao University, Qingdao, China

**Keywords:** lipoprotein lipase, hypertriglyceridemia, mutation, acute pancreatitis, compound heterozygosity

## Abstract

Lipoprotein lipase (LPL) is widely expressed in skeletal muscles, cardiac muscles as well as adipose tissue and involved in the catabolism of triglyceride. Herein we have systematically characterized two novel loss-of-function mutations in LPL from a Chinese family in which afflicted members were manifested by severe hypertriglyceridemia and recurrent pancreatitis. DNA sequencing revealed that the proband was a heterozygote carrying a novel c.T928C (p.C310R) mutation in exon 6 of the LPL gene. Another member of the family was detected to be a compound heterozygote who along with the c.T928C mutation also carried a novel missense mutation c.A1187T (p.E396V) in exon 8 of the LPL gene. Furthermore, COS-1 cells were transfected with lentiviruses containing the mutant LPL genes. While C310R markedly reduced the overall LPL protein level, COS-1 cells carrying E396V or double mutations contained similar overall LPL protein levels to the wild-type. The specific activity of the LPL mutants remained at comparable magnitude to the wild-type. However, few LPL were detected in the culture medium for the mutants, suggesting that both mutations caused aberrant triglyceride catabolism. More specifically, E396V and double mutations dampened the transport of LPL to the cell surface, while for the C310R mutation, reducing LPL protein level might be involved. By characterizing these two novel LPL mutations, this study has expanded our understanding on the pathogenesis of familial hypertriglyceridemia (FHTG).

## INTRODUCTION

Severe hypertriglyceridemia refers to fasting triglyceride exceeding 1000 mg/dL (11.3 mmol/L) [1]. At that level, chylomicrons (carrying dietary fat) start to accumulate in the serum, causing multiple attacks of acute pancreatitis [2, 3]. On top of extremely elevated triglyceride concentrations, clinical sequelae of chylomicronaemia involved eruptive xanthomas, lipaemia retinalis, recurrent epigastric pain or acute pancreatitis and hepatosplenomegaly [4, 5]. Severe hypertriglyceridemia can be divided into primary and secondary groups. Primary hypertriglyceridemia is mainly caused by loss-of-function genetic defects leading to blunted lipolysis of chylomicrons and thus accumulation of triglyceride. These mutations are mostly concentrated in lipoprotein lipase (LPL) which catabolize triglyceride in non-hepatic tissues, apolipoprotein C-II (APOC2) which acts as an essential LPL activator, apolipoprotein A-V (APOA5) which stabilize the lipoprotein–LPL complex, lipase maturation factor 1 (LMF1) which is involved in the folding and expression of LPL, and glycosylphosphatidylinositol-anchored high density lipoprotein-binding protein 1 (GPIHBP1) which mediates the transmembrane transport of LPL and the binding between lipoprotein and LPL [6, 7]. Among them, LPL defects with autosomal recessive transmission accounts for more than 90% of FHTG [8]. On the other hand, secondary hypertriglyceridemia can be derived from obesity, poorly controlled diabetes, excessive alcohol consumption, hypothyroidism, nonalcoholic fatty-liver disorder, renal failure, nephrotic syndrome and other rare metabolic disorders [9].

The human LPL gene maps on chromosome 8p22 and consists of ten exons [10]. Human LPL protein is a noncovalent homodimer with a head-to-tail configuration. It exhibited two domains: a large N-terminal domain (amino acid residues 1–315) and a small C-terminal domain (amino acid residues 316–448) [11]. C-terminal domain promoted the formation and stability of the LPL homodimer [12, 13]. It also enhanced the expression of LPL activity, due to the fact that dissociation of LPL homodimer leaded to loss of activity [13]. Until now, 114 mutations in the LPL gene have been identified to induce chylomicronaemia, of which a majority of missense mutations cluster in the highly conservative exons 4, 5 and 6 [14]. Since all the attempts to characterize the 3-D structure of human LPL have failed so far, site-directed mutagenesis remains to be the primary resort for studying the genotype-phenotype spectrum of LPL. Nonetheless, the 3-D structure of the highly homologous pancreatic lipase has afforded some valuable hints [15].

LPL is predominantly synthesized by skeletal muscles, cardiac muscles and adipose tissues [16]. It is generally believed that LPL is secreted into the interstitial fluid and then transported across capillary endothelial cells and anchored on the lumen surface of capillaries with the assistance of GPIHBP1 [17]. LPL hydrolyzed triglyceride from circulating chylomicrons and very-low-density lipoprotein (VLDL), liberating free fatty acids for energetic metabolism or storage in adipose tissues [18]. Intravenous injection of heparin can strip LPL from the surface of endothelial cells so that the LPL activity can be measured [19].

In this study, we identified a Chinese family manifested by severe hypertriglyceridemia due to primary LPL deficiency. The proband turned out to be a heterozygous carrier for a novel missense T928C substitution (p.C310R) in exon 6 of the LPL gene, and another member of the family was found to be a compound heterozygote for the T928C and a A1187T mutation in exon 8(p.E396V). The effect of these mutations on LPL function was then examined in cell culture. By characterizing the two novel LPL mutations, we aimed to shed new insight on the pathogenesis of FHTG.

## RESULTS

### Laboratory data of heterozygous family members and treatment

**Figure 1:**
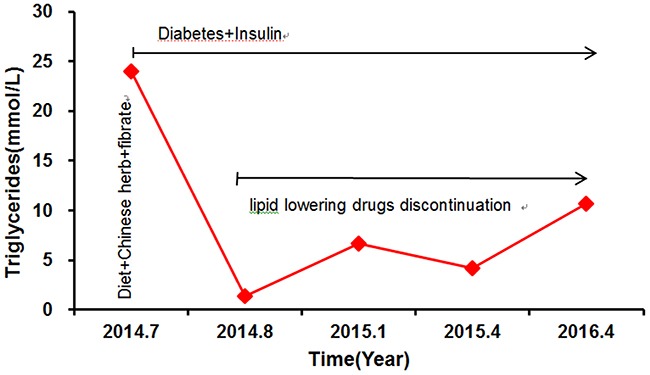
The proband's triglyceride levels and response to treatment Aspart 12 IU was administrated three times a day before each meal and Glargine 20 IU was administrated before sleep. Fenofibrate, 100mg three times a day.

The proband MJH's response to treatment with lipid-lowering medications between 2014 and 2016 was illustrated in Figure 1. Plasma TG showed large fluctuations during that period. He exhibited extremely high concentrations of fasting triglyceride (23.97 mmol/L) on the occasion of our first evaluation, which was 12 times higher than the upper normal range. The levels of total cholesterol (TC), low-density lipoprotein (LDL-C), and high-density lipoprotein (HDL-C) were 8.30 mmol/L, 5.64 mmol/L, and 0.75 mmol/L (Table 1), respectively. After hospitalized, he was subjected to stringent low-fat diet, Chinese herb, and Fenofibrate (100 mg three times a day), as well as intensive insulin therapy (Aspart 12 IU was administrated three times a day before each meal, Glargine 20 IU was administrated before sleep), to achieve glucose and lipid metabolism balance (Figure 1). Upon administration of lipid-lowering medications, he exhibited a much-improved lipid profile with TG level as low as 1.33 mmol/L (Figure 1). Due to the lack of symptoms, he was refractory to stick to low-fat diet and antihyperlipidemic treatment. In consequence, he presented with a subsequent relapse of hypertriglyceridemia (TG: 6.67 mmol/L) combined with relatively low LDL (2.34 mmol/L) and HDL (0.8 mmol/L) six month after being discharged from hospital (Figure 1). Since then, his TG levels displayed a stepwise increased tendency because of discontinuation of antihyperlipidemic drugs. The following two evaluations of TG levels were 4.16 mmol/L and 10.65 mmol/L, respectively (Figure 1).

**Table 1:** Lipid biochemistry of the proband and his family members

ID	TG (mmol/L)	TC (mmol/L)	HDL (mmol/L)	LDL (mmol/L)	Lp(a) (mg/L)	FFA (mmol/L)
I-2	23.97	8.30	0.75	5.64	363	—
II-1	22.20	16.34	0.5	1.7	398	0.96
I-1	2.59	4.19	1.15	2.49	—	—
I-3	3.13	5.51	1.09	3.57	—	—
III-1	0.87	3.20	1.22	1.75	—	—
Normal range	0.3-1.92	2.32-5.62	0.8-1.8	1.9-3.12	0-300	0.1-0.45

TG, triglyceride; TC, total cholesterol; HDL, high-density lipoprotein; LDL, low-density lipoprotein; Lp(a), lipoprotein(a); FFA, free fatty acid; —, not tested.

**Figure 2:**
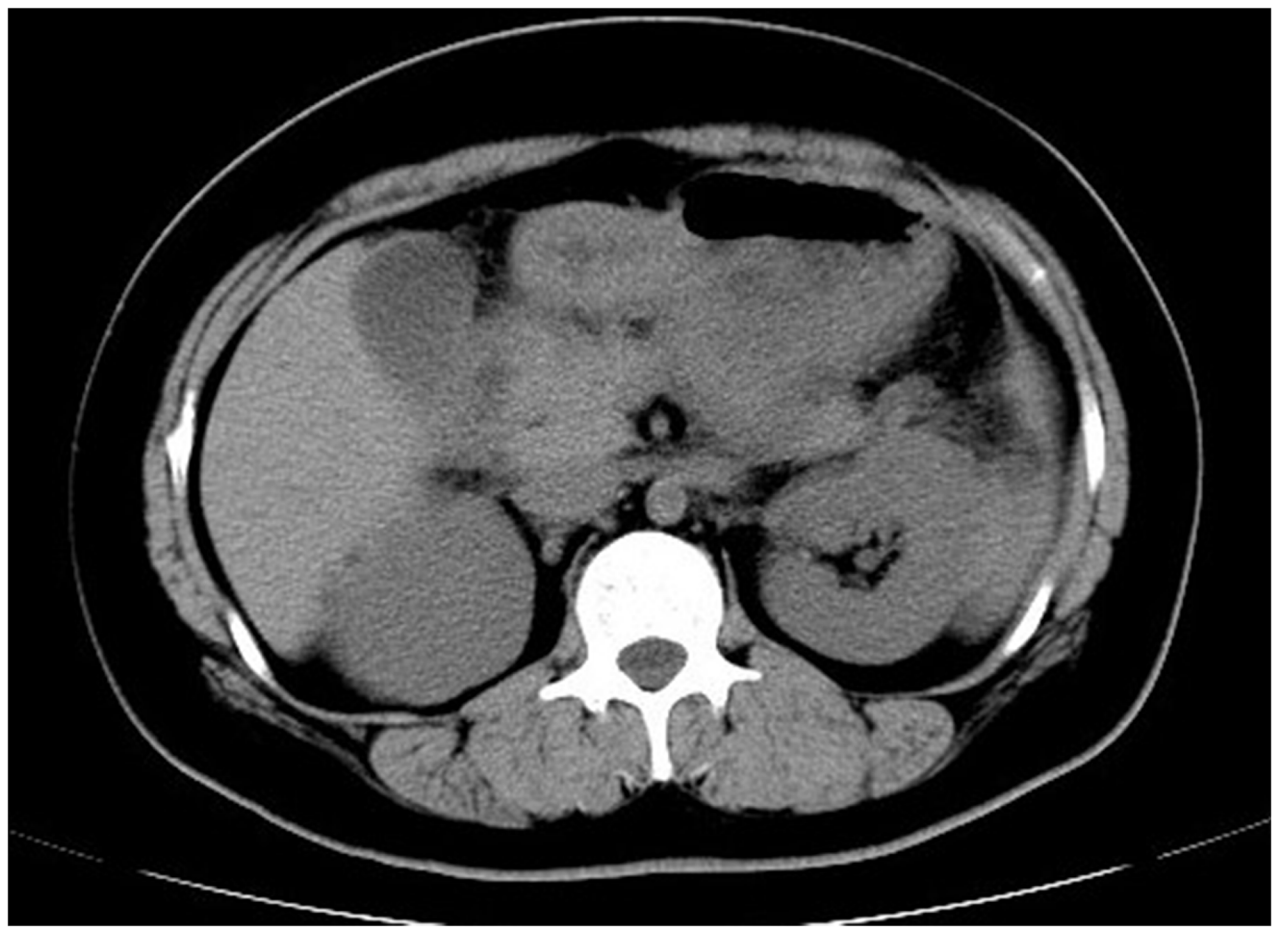
Abdominal computed tomography A typical computed tomography manifestation of enlarged pancreas with blurred outline, disappearance of peripancreatic space and thickened left renal fascia was highly suggestive of acute pancreatitis.

The proband's niece, MC (II-1), carried more severe hyperlipidemia. She was admitted with symptoms of acute pancreatitis and deleterious lipid profile as evidenced by abnormal plasma concentrations of TG (22.20 mmol/L) and TC (16.34 mmol/L) but low HDL (0.5 mmol/L) and LDL (1.7 mmol/L) (Table 1). The typical computed tomography manifestation further supported the diagnosis of acute pancreatitis (Figure 2). In the context of abdominal pain and hypertriglyceridemia, MC was placed on cessation of oral intake, antacid and alimentary replacement. After the treatment, the plasma triglyceride level decreased to 4.94 mmol/L, but C-reactive protein was still high (26.02 mg/L). She was then prescribed with Chinese herb, Fenofibrate (0.1 g bid), antacid, and a regime of liquid low-fat diet. Her serum triglyceride concentrations still fluctuated, ranging from 6 mmol/L to 8 mmol/L. After amelioration of acute pancreatitis, she was discharged with a prescription of lipid-lowering drugs mentioned previously. However, she still experienced multiple attacks of acute pancreatitis upon digestion of a paucity of red meat, which is a strong indication of genetic deficiency.

Lipid biochemistry of 4 hypertriglyceridemic family members (I-2, II-1, I-1 and I-3) and 10 normolipidaemic controls was shown in Table 2.

**Table 2:** Lipid biochemistry of controls and HTG (hypertriglyceridemic) subjects

	Total	TG (mmol/L)	TC (mmol/L)	HDL (mmol/L)	LDL (mmol/L)
HTG	4	10.55±11.49*	7.51±5.30	0.94±0.31	3.03±1.64
Controls	10	0.57±0.19	4.52±0.79	1.25±0.40	2.05±0.50

The parameters are presented as mean±SD. TG levels were log-transformed before analysis. *, P< 0.05, significant differences from controls. TG, triglyceride; TC, total cholesterol; HDL, high-density lipoprotein; LDL, low-density lipoprotein.

### Genetic analysis

DNA samples from the proband MJH and his niece MC were extracted for whole-exome sequencing, which revealed two novel mutants, C310R and E396V, in LPL gene. Analysis of proband's DNA sequence indicated a heterozygous c.T928C (p.C310R) mutation in exon 6 of the LPL gene, which converted a cysteine codon into an arginine codon. As for MC, she was detected to be a compound heterozygote who along with the c.T928C mutation also carried a novel missense mutation c.A1187T (p.E396V) in exon 8 of the LPL gene. The latter missense mutation entailed protein conversion from glutamic acid to valine. Both two mutations have already been submitted to NCBI clinical variation (SCV000262583, SCV000262584). Not only were loss-of-function mutations in LPL gene detected but a few of single nucleotide polymorphisms (SNPs) in LMF1 (rs142481016), GPIHBP1 (rs11538389), and APOA5 (rs201229911) genes were also noted. Nevertheless, according to the recent genome-wide association study, none of these SNPs is associated with the elevated triglyceride concentrations [20]. Importantly, on top of the aforementioned meaningless substitutions, there was no functional variants existed in APOC2, APOA5, GPIHBP1, and LMF1 genes, which was known to have causative effect on monogenic hypertriglyceridemia [6, 7, 21–24]. The genotype of APOE was E3/E3 for both subjects.

**Figure 3:**
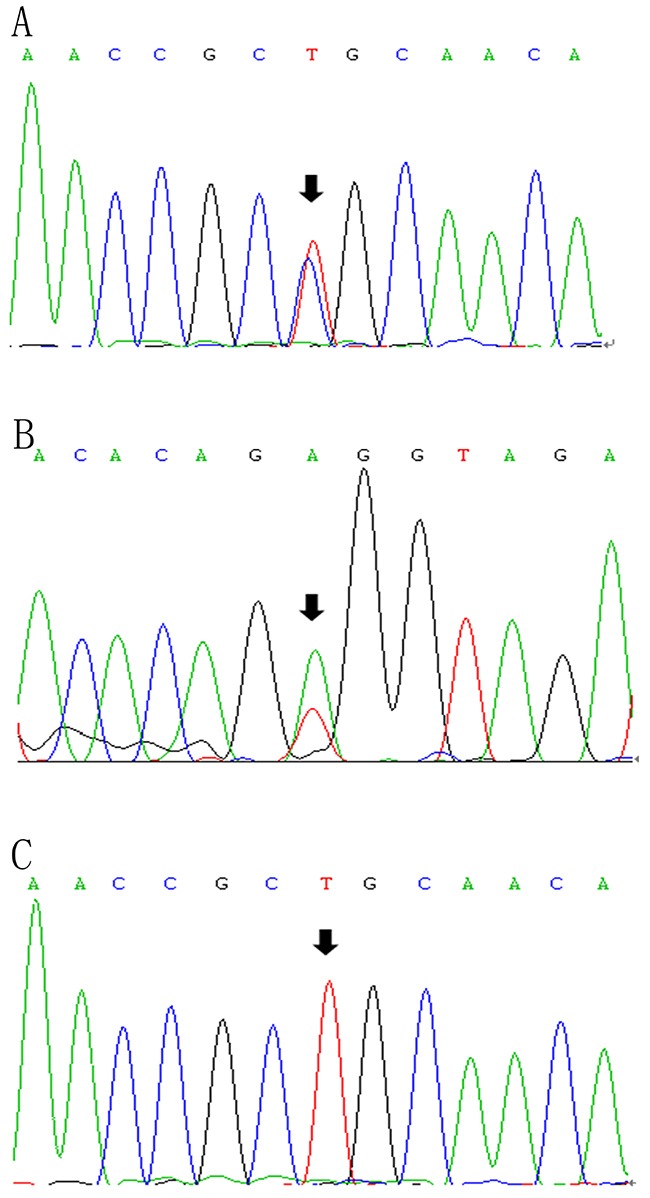
LPL gene (reference sequence NM_000237)sequencing diagram The diagram showed the partial forward sequence of exon 6 in the proband **(A)**, a control subject **(C)** and reverse sequence of exon 8 in the proband's niece **(B)**.

Followed by whole-exome sequencing, DNA samples from 19 family members (including the proband) were amplified to identify frequency of mutations in the family. Figure 3 represents the partial sequence diagram of the LPL gene. Two novel mutations were also screened in 100 normolipidemic subjects, which showed normal nucleotide sequence without any variation, proving that neither of the mutations was SNP.

### Analysis of LPL activity and mass in plasma

**Figure 4:**
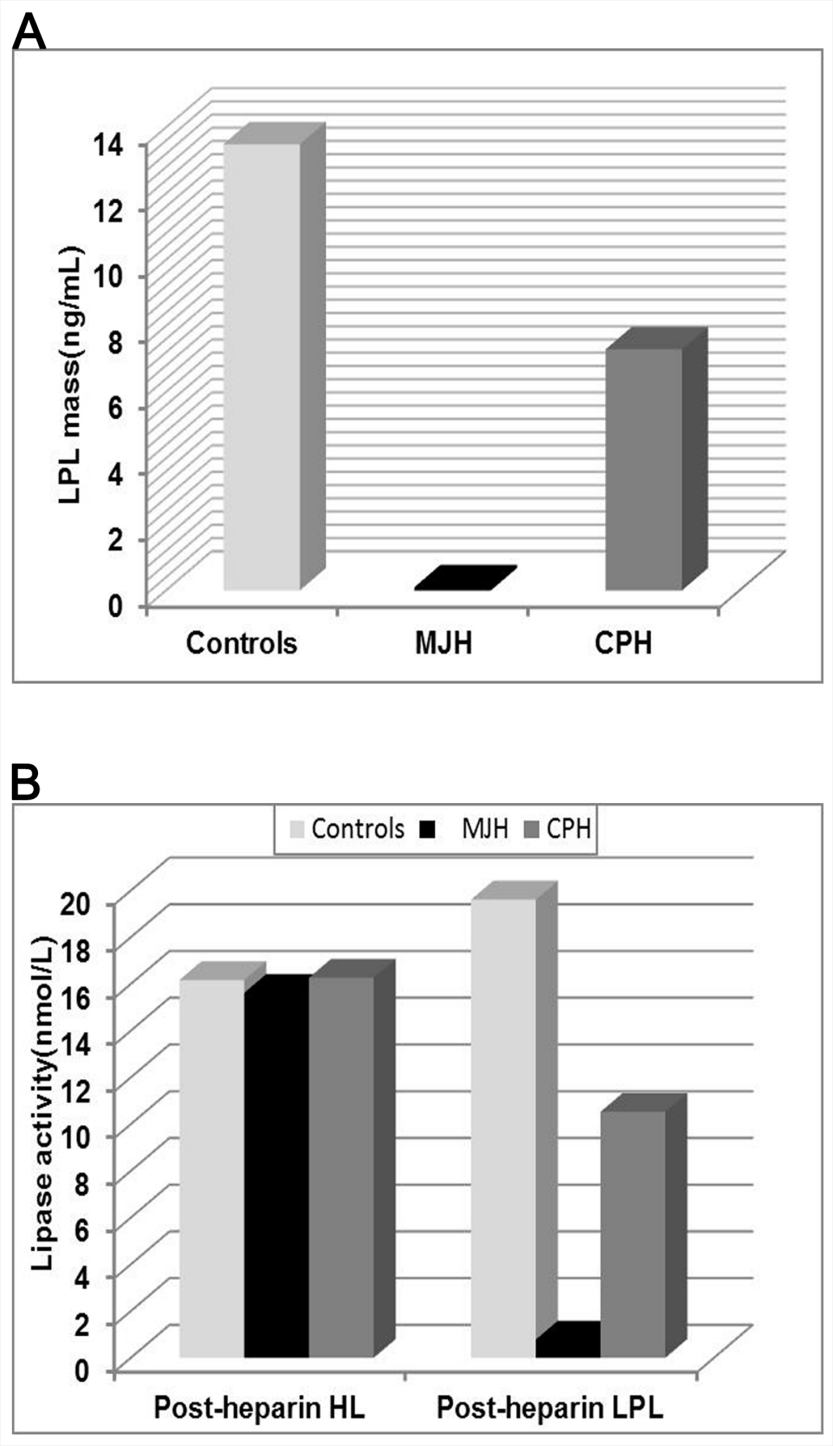
Analysis of LPL activity and mass in plasma The post-heparin LPL mass **(A)** and lipase activity **(B)** was measured in the proband MJH, CPH (I-1) and 10 normolipidemic subjects. Peripheral blood was collected at 10 min after heparin injection (60IU/kg) to assay lipase activity and LPL mass by enzyme-fluorescent method and ELISA, respectively. The data of controls were presented as mean.

Post-heparin lipase activity and LPL mass of proband MJH, CPH (I-1), and 10 normolipidemic controls were measured by ELISA and enzyme-fluorescent method. Post-heparin assay was not carried out in the proband's niece MC because of her vulnerable vessel wall inapplicable for injection. In contrast to normolipidemic controls (13.49 ng/mL), the level of post-heparin LPL mass in proband MJH (0.1 ng/mL) was dramatically reduced by 135 times (Figure 4). As for CPH, the level of LPL mass (7.29 ng/mL) was nearly half the mean value of the controls (13.49 ng/mL) (Figure 4). Correspondingly, post-heparin LPL activity in MJH (0.78 nmol/mL) and CPH (10.53 nmol/mL) virtually displayed the same pattern as LPL mass in comparison with the controls (19.61 nmol/mL) (Figure 4). Alluding to hepatic lipase (HL) activity, the differences among MJH, CPH, and controls seemed negligible, which indicated that an intact hydrolyzing action of hepatic lipase was presented in MJH and CPH. Consequently, LPL C310R and E396V mutations may be responsible for defective triglycerides metabolism leading to extremely elevated serum triglycerides.

### Analysis of LPL dimerization in plasma

**Figure 5:**
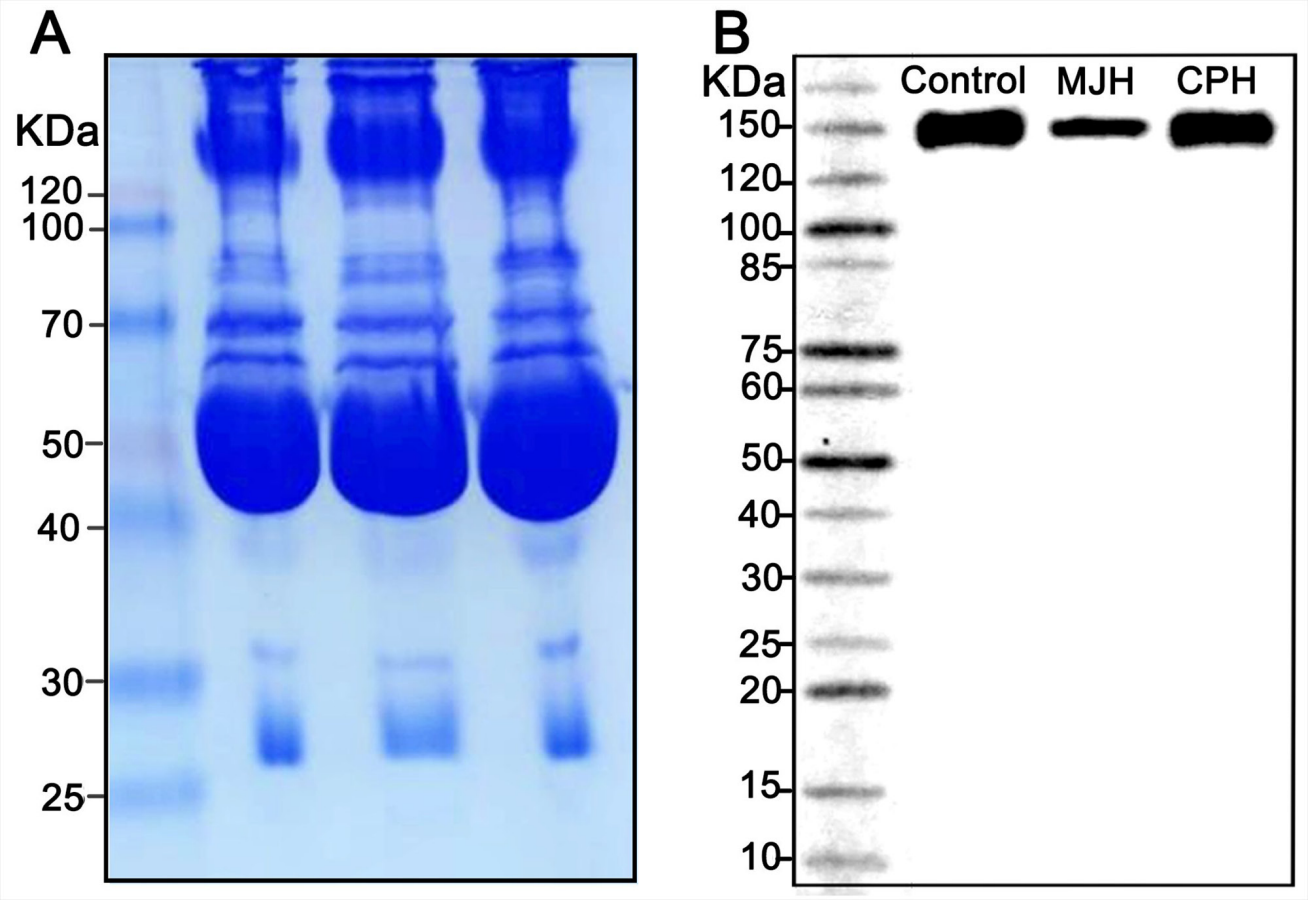
A native gel western blot analysis of LPL dimerization in post-heparin plasma Coomassie staining was used as a loading control **(A)**. Post-heparin plasma of the proband MJH, CPH(I-1), and a normolipidemic control was analyzed by a native gel western blot **(B)**.

To elucidate whether C310R and E396V mutations suppressed the dimerization of LPL, post-heparin plasma of the proband MJH, CPH, and a normolipidemic control was analyzed by a native gel western blot. The result revealed that both mutations exerted no influence on the dimerization of LPL (Figure 5).

### Functional analysis of LPL mutants *in vitro*

#### Relative expression of mRNA

**Figure 6:**
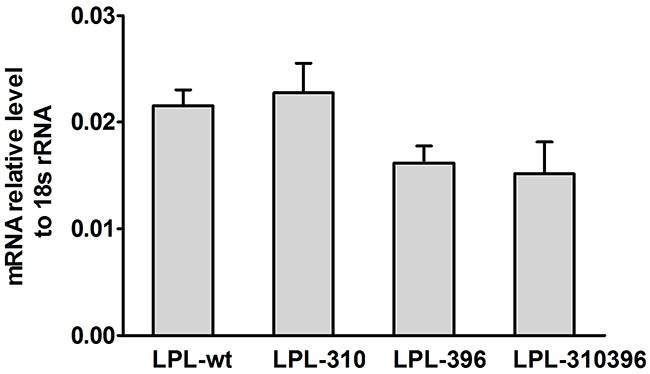
Quantitation of extracted mRNA and normalized to 18S rRNA mRNA was extracted from transfected COS-1 cells containing the mutant LPL genes and quantitatively determined by qPCR. The mRNA levels of LPL-310, LPL-396, and LPL-310396 were almost equal to that of LPL-wt, and there was no significant difference between the wild-type and mutants (P>0.05). Values are shown as mean±SD. Each experiment was repeated three times.

mRNA was extracted from transfected COS-1 cells and analyzed by fluorescent RT-PCR. The result indicated that the amount of LPL-310, LPL-396 and LPL-310396 was similar to that of the LPL wild-type (LPL-wt) (P >0.05) (Figure 6), suggesting limited effect of LPL mutants on the level of LPL gene transcription.

#### Analysis of LPL activity and mass

**Figure 7:**
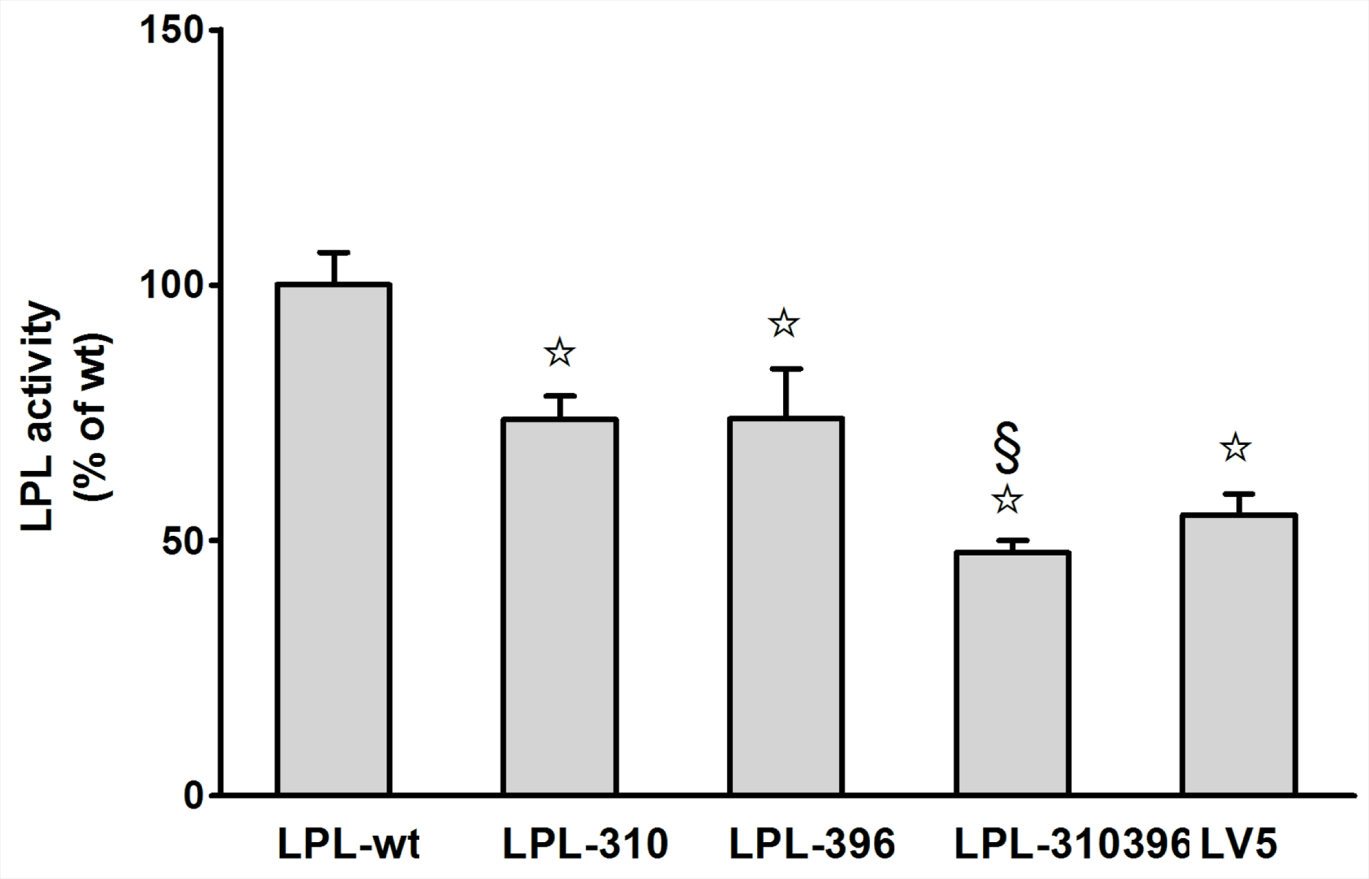
Functional analysis of LPL mutants in the medium Activity of LPL mutants was assayed as a percentage of LPL wild-type after transfection. Each experiment was repeated by measuring six separate dishes. Values represent the mean ±SEM. ☆and §, significant (P<0.05) differences from LPL-wt, LPL-310, respectively.

**Figure 8:**
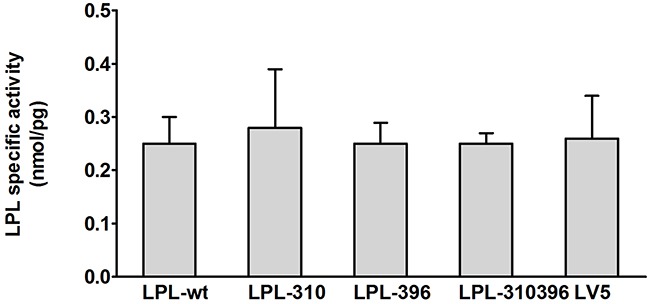
Functional analysis of LPL mutants in the cell lysates Specific activity of LPL in the cell lysates is calculated by dividing LPL activity by LPL mass. There was no significant difference between the wild-type and mutants (P>0.05). Values are shown as mean±SD.

Functional significance of LPL mutants was confirmed by analyzing LPL activity and mass in cell culture medium and cell lysates via enzyme-fluorescent method and ELISA, respectively. The data showed that LPL-310, LPL-396, and LPL-310396 produced noticeably decreased LPL activity, accompanied by undetectable LPL mass in the medium compared to LPL-wt (P<0.05) (Figure 7). Interestingly, the activity of LPL-310396 was two times lower than that of LPL-wt (Figure 7), indicating that negligible mutant LPL was released by heparin (Table 3). Of note, the activity and mass of LPL-310396 resembled to those of LV5 (blank vector) (P>0.05). It indicated that the release of LPL-310396 was not induced by heparin due to disrupting conformational stability. Intriguingly, the activity and concomitant mass of LPL-310396 in the cell lysates was nearly two times higher than that of LPL-wt (P<0.01) (Table 3), which is opposed to the above finding in the medium. It may suggest that LPL-310396 is not secreted due to dysfunction of intracellular LPL protein trafficking that leads to accumulation of the proteins in the cells. In addition, the activity and mass of LPL-396 in the cell lysates was slightly higher than that of LPL-wt (P<0.05). In contrast, the activity and mass of LPL-310 was lower than that of LPL-wt (P<0.05). Although the LPL activity in the cell lysates increased continuously with increasing LPL mass, there was no significant difference between the activity of LPL-wt and LPL mutants in the cell lysates after adjusting the effect of LPL mass on LPL activity (Figure 8). These data suggested that the E396V mutation might impair transport of LPL to the cell surface and C310R reduced overall LPL protein level.

**Table 3:** The activity and mass of LPL wild-type and mutants in transfected COS-1 cells

Plasmids (n=6)	Medium	Cell lysate		Specific activity	
	Activity(nmol/mL)	Mass(pg/mL)	Activity(nmol/mL)	Mass(pg/mL)	(nmol/pg)
LPL-wt	2.71±0.42	15.51±3.25	10.10±2.15	40.46±2.87	0.25±0.05
LPL-310	2.00±0.31*	ND	7.09±2.16*	24.61±2.33***	0.28±0.11
LPL-396	2.00±0.64*	ND	12.25±1.71*	50.76±4.29*	0.25±0.04
LPL-310396	1.29±0.16***	ND	15.17±1.53***	58.16±4.33***	0.25±0.02
LV5	1.49±0.24***	ND	4.31±1.32***	17.19±3.35***	0.26±0.08

The plasmids of LPL wild-type, LPL-310, LPL-396, LPL-310396 and LV5 were transfected into COS-1 cells. Medium and cell lysates were collected after 20 units/mL heparin incubation. LPL activity and mass in the medium and cell lysates were quantified by enzyme-fluorescent method and ELISA, respectively. Detection limits of LPL activity and mass are 0.03 nmol/mL and 5 pg/mL, respectively. Each experiment was repeated by measuring six separate dishes. The activity and mass values are shown as mean±SD. ND, not detectable. *, P<0.05.***, P<0.01, significant differences from LPL wild-type. Specific activity is calculated by dividing LPL activity by LPL mass in the cell lysates.

## DISCUSSION

In this study, we systematically characterized two novel loss-of-function mutations in LPL from a Chinese family in which afflicted members (except MC's daughter) were manifested by mild to severe hypertriglyceridemia and recurrent pancreatitis. This familial aggregation phenomenon and noxious lipoprotein disturbances prompted us to disentangle this puzzle by determining the LPL activity and mass in post-heparin plasma and by analyzing the LPL genetic defect *in vitro*. Our data demonstrated that the proband MJH and his sister MXH (I-3) were heterozygotes and both harbored c.T928C mutation in exon 6 of the LPL gene which resulted in conversion of cysteine to arginine (p.C310R). Notably, the proband's niece MC was a compound heterozygote who along with the c.T928C mutation also carried a novel missense mutation c.A1187T in exon 8 of the LPL gene. The latter missense mutation induced the replacement of glutamic acid with valine (p.E396V). Furthermore, COS-1 cells were transfected with lentiviruses containing the mutant LPL genes. *In vitro* studies, the data revealed that the global amount of LPL-310 synthesized in COS-1 cells was markedly reduced compared to LPL-wt (Table 3). However, the amount of LPL-310 mRNA was similar to that of LPL-wt, indicating that C310R mutation barely affected the stability of transcription but tremendously suppressed post-transcriptional modification of the LPL gene. This mutation led to decreased secretion of LPL from COS-1 cells and concomitantly compromised LPL activity upon heparin treatment. On the contrary, the total amount of both LPL-396 and LPL-310396 synthesized was almost equal to that of LPL-wt, which is in line with previous identical mRNA expression. Therefore, the reason why lower LPL-396 and LPL-310396 reached into medium can be interpreted by that the E396V mutation dampened intracellular LPL trafficking, which resulted in extremely low LPL transported to the surface of COS-1 cells and nearly undetectable LPL activity. Thus, LPL accumulated within the COS-1 cells in large quantities. Of note, the activity of LPL mutants depended on the change of LPL mass in the cell lysates, as evidenced by the result that there was no significant difference between activity of LPL wild-type and LPL mutants in the cell lysates after eliminating the effect of LPL mass on LPL activity (Figure 7).

It becomes evident that normal LPL is required to bind to heparin in order to separate from parenchymal cells and release into extracellular fluid where LPL is transported across the capillary endothelial cells in the presence of GPIHBP1 [25]. Heparin binding sites for LPL are located at residues 279–282 and 292–304 in the N-terminal region, as well as Lysine-319, Lysine-403, Arginine-405, Lysine-407 and Lysine-413–414 in the C-terminal region [26]. In the present study, our newly identified mutation Arg-310 was adjacent to the heparin-binding sites in the N-terminal region and so is Valine-396 in the C-terminal region. Furthermore, the C310R mutation in exon 6 resulted in the replacement of electrically neutral cystine with positively charged arginine. Also, the E396V mutation in exon 8 resulted in the conversion of negatively charged glutamic acid to electrically neutral valine. Both mutations are believed to disrupt conformational stability of LPL through electrical charge transfer. Taken together, the evidence points to the possibility that electrical charge transfer in the vicinity of the heparin-binding sites compromised the conformational stability of LPL and induced a marked reduction in the affinity of LPL to heparin. That seemed to explain the findings that all the mutant LPL exhibited scarcely detectable LPL activity after incubation with heparin. Buscà *et al*. discovered that the C-terminal region of LPL promoted post-translational handling of LPL in the rough endoplasmic reticulum (rER) and that truncated LPL (F388→Stop LPL) was not capable of being efficiently secreted from COS-1 cells and therefore accumulated inside the rER [27]. Consistent with these findings, LPL-396 and LPL-310396 might have difficulty to depart from rER due to mutated C-terminal region and thus cause the accumulation of mutant LPL inside the COS-1 cells. This mutation (E396V) demonstrated the importance of the C-terminal region of the protein in the regulation of LPL expression and activity. Yaomin Hu *et al*. reconstructed the three-dimensional model structure of the human LPL using the UCSF Chimera package, which was based on the known crystal structure of pancreatic lipase [28]. They identified Lys312insC and Thr361insA mutations in the LPL gene, both of which led to a truncation of the whole or part of C-terminal domain and would jeopardize the stability of the LPL dimer. These two mutations are believed to cause significant reduction of LPL activity by impairing LPL homodimerization [28]. But our findings conflicted with that conclusion, the newly identified E396V mutation exerted no influence on the dimerization of LPL. The reason may lie in the fact that E396V point mutation simply resulted in one amino acid conversion in comparison with considerable shortening of the C-terminal domain of LPL.

Most LPL missense mutations have been identified in exon 4, 5 and 6 (residues 117-312). Yet, a few studies were concerned with mutations in exon 8 including W382X [29], W394X [30], K434N [31] and G436R [31], which surmised that these mutations were related to severe hypertriglyceridemia. Nevertheless, these studies just scratched the surface of the subject and thus we cannot draw a definitive conclusion from it. In contrast, our newly identified E396V mutation was conclusive of having a negative impact on triglyceride metabolism *in vitro* study.

We have already set up C57BL mouse models of hypertriglyceridemia expressing the C310R mutation. Additional studies are being carried out to confirm the role of defective LPL action on triglyceride metabolism in these animal models.

In conclusion, we identified two novel pathogenic missense mutations in the LPL gene (C310R/E396V) as causes for primary hypertriglyceridemia. Based on the fact that the three dimensional structure of LPL is still not available, the only access to unveil its mask is to deduce LPL conformational change by investigating the relationship between LPL gene mutations and its functional significance.

## MATERIALS AND METHODS

### Subjects

**Figure 9:**
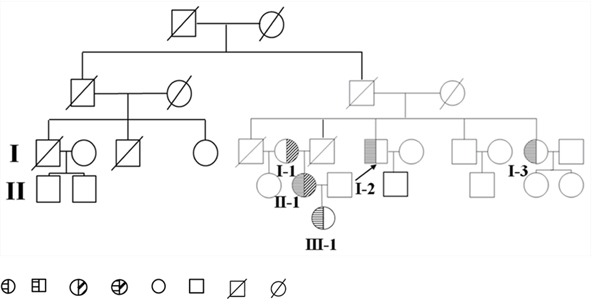
Pedigree diagram of the family Proband is shown by the arrow. hypertriglyceridemia subjects who are heterozygous for the mutant C310R LPL gene. Hypertriglyceridemia subjects who are heterozygous for the mutant E396V LPL gene. Hypertriglyceridemia subject who is compound heterozygous for the mutant C310R and E396V LPL gene combined. Normolipidemic subjects. Deceased subjects.

The proband, a 48-year-old Chinese male, was admitted to our hospital in 2014 for persistent elevation of glucose (fasting blood glucose at 12.25 mmol/L, HbA1c at 9.7%, ketone at 1+). After initial diagnosis with type 2 diabetes in 2007, he was prescribed with metformin and insulin Aspart-30 injections. However, the control of his blood glucose remained unideal over the years due to poor compliance in diet and exercise. During the latest admission, his plasma TG, TC and LDL-C were elevated to 23.97 mmol/L, 8.30 mmol/L and 5.64 mmol/L respectively (Table 1). Thyroid, liver and renal functions were within the normal range. Physical examination was unremarkable (BMI: 25 kg/m^2^) without significant signs of abdominal pain, hepatosplenomegaly, eruptive xanthoma and lipaemia retinalis. He reported recurrent episodes of acute pancreatitis for four times from 2002 to 2005 after alcohol intake or red meat consumption. His family history revealed the possible presence of hereditary hypertriglyceridemia with an autosomal recessive pattern (pedigree shown in Figure 9). Of note, his sister MXH (I-3) and niece MC (II-1) both presented with recurrent episodes of pancreatitis previously. MC developed pancreatitis since the age of seventeen, characterized by constant lipoprotein disturbance and poor response to lipid-lowering drugs (Fenofibrate and Chinese herbs). None of the family members had history of autoimmune disease, gallstones or drug abuse. The proband had a history of drinking liquor (up to 500 mL) and smoking cigarettes (up to 40) per day for thirty years.

Ten healthy volunteers (48.6 ± 4.0 years old) were studied as controls, including 5 male subjects (50.6 ± 4.4 years old) as the age-adjusted controls for MJH, and 5 females (46.6 ± 2.7 years old) for CPH (I-1). Informed consent was obtained from all subjects investigated and the study protocol had the approval of our local ethics committee.

### Plasma lipid analysis

Blood samples of 19 family members (including the proband) were collected after an overnight fast, and then examined for thyroid, liver, renal function, autoimmune disease. Lipid profile including plasma TG, TC, LDL, HDL-C, free fatty acid (FFA), lipoprotein(a)(Lp(a)) was determined by enzymatic methods on a 7600 series automatic analyzer (Hitachi Limited, Japan).

### Genetic analysis of the family

Genomic DNA was extracted from peripheral blood cells by a Blood DNA kit (Aidlab Biotechnologies, Beijing, China). The DNA samples of MJH and MC (1 μg each) were captured by an Agilent SureSelect Human All Exon V5/V6 Kit (Novogene, Beijing, China) followed by whole-exome sequencing on a HiSeq 4000 Sequencer (Novogene, Beijing, China). A number of candidate genes involved in lipid metabolism, including LPL, APOC2, APOA5, LMF1, GPIHBP1 and apoprotein E (APOE), were examined.

To verify the frequency of mutations screened out by next-generation sequencing (LPL:exon6:c.T928C:p.C310R and exon8:c.A1187T:p.E396V) in the family, an aliquot of 2 μL DNA from each family member, 12.5 μL 2×Taq PCR MasterMix (Aidlab Biotechnologies) and 1.5 μL primers (10 μmol/L) was included in the PCR system. The amplification conditions for both exons were 95 °C for 3 min, denaturation at 95 °C for 30 s, annealing at 57 °C for 30 s, and extension at 72 °C for 45 s for a total of 38 cycles and last extension step at 72 °C for 10 min. PCR product was afterwards directly sequenced by first-generation sequencing (Sangon Biotech, Shanghai, China). Primers for LPL exon 6 and exon 8 were listed below [32]:

Exon-6F 5’-AGAGGATCCTTCTGCCGAGATACAATCTTGGTGTC-3’

Exon-6R 5’-AGGCTGCAGGACTCCTTGGTTTCCTTATTTACAACA-3’

Exon-8F 5’-GCCGGATCCGATCTCTATAACTAACCAAATTTATTGCT-3’

Exon-8R 5’-TCCCTGCAGTGGGGGTCTAAAGTGAAGGAAGAAAA-3’

### Determination of LPL activity and mass in plasma

To determine LPL activity and mass, intravenous injection of heparin was performed to strip LPL from the surface of endothelial cells [19]. Briefly, after fasting overnight, heparin (60 IU/kg body weight) was injected, and peripheral blood was collected at 10 min after injection. To rule out interfering effect derived from medication, the subject stopped insulin treatment on the same morning. Blood samples were collected in EDTA tubes and placed on ice immediately. Then plasma was separated and LPL mass was measured by a Human LPL ELISA kit (TSZ Biological Trade, New Jersey, USA).

An LPL activity assay kit (Roar biochemical, NY) was employed for the determination of total lipase activity including both LPL and HL. To correct for the contribution from HL, 1M NaCl was added and incubated at 37 °C for 60 min, so that the LPL activity can be completely inhibited.

### Analysis of LPL dimerization in plasma

Briefly, equal volumes of post-heparin plasma samples were diluted 1:10 and mixed with non-denatured loading buffer. Proteins were separated by Native-PAGE (8% acrylamide gel, 150 V, 90 min) and transferred onto polyvinylidene difluoride (PVDF) membranes (200mA, 120min then 300mA, 30min). Membranes were incubated overnight at 4°C with a mouse anti-LPL antibody (1:1000, Abcam) after blocking with 5% dry milk in Tris buffered saline-Tween (TBST). After being washed 3 times for 15 minutes with TBST, membranes were incubated 1 hour with HRP-conjugated secondary antibodies. After incubation with chemiluminescent HRP substrate (Millipore Corporation, Billerica, USA), bands can be visualized. Coomassie staining was used as a loading control.

### Functional analysis of LPL mutants *in vitro*

#### Construction of lentiviruses containing site-directed mutagenized LPL cDNA

pCMV6-XL5 vector containing normal human LPL cDNA (Origene, America) was used as a template to generate site-directed mutagenesis. The C310R, E396V and C310R/E396V double mutations were introduced into LPL cDNA by mutagenic primers by a KOD-Plus-Mutagenesis Kit (Toyobo, Osaka, Japan). The LPL^C310R^, LPL^E396V^, LPL ^C310R/E396V^ along with LPL wild-type cDNA were cleaved out of the original vector, and cloned into a lentiviral vector LV5 (CMV-MCS-EF1-PURO). Four LV5 vectors containing the wild-type and mutant LPL cDNA sequences, along with blank LV5 vector as the background control, were transfected into 293T cells by Lipofectamine 2000 (Invitrogen, Carlsbad, CA) with the assistance of a Lenti-Easy Packaging Mix (Genechem, Shanghai, China). After 48-72 h, cell medium was collected and lentivirus was harvested by ultracentrifugation to a titration at 1 × 10^8^ TU/mL. The multiplicity of infection (MOI) value of these lentiviruses was measured to be 50 (Supplementary Figure 1).

#### Lentivirus transfection

COS-1 cells were obtained from American Type Culture Collection (ATCC) and maintained in Dulbecco's modified Eagle's medium (DMEM, Hyclone, Logan, UT, USA) supplemented with 10% fetal bovine serum (Hyclone, Logan, UT, USA) at 37 °C in a 5% CO_2_ environment. COS-1 cells were plated at 3×10^4^ per well into six-well plates and cultured overnight. Culture medium was then replaced and 50 μL lentiviruses was added along with 2 μL polybrene (5 μg/mL) (GenePharma, Shanghai, China). After culturing for 24 h, the transfected COS-1 cells were washed with phosphate buffer saline (PBS) and cultured in fresh DMEM medium. GFP fluorescence started to blow from 3 days after transfection. Subsequently, transfected COS-1 cells were incubated in puromycin-containing(7 μg/mL) DMEM medium to select for stably transfected cells.

#### Real-time quantitative PCR

Transfected COS-1 cells (1 × 10^7^) were collected and RNA was extracted by an EASYspin RNA Mini Kit (Aidlab Biotechnologies, Beijing, China). To assess the effect of LPL mutants on transcription, the first-strand cDNA was obtained by reverse transcription with random primer (0.1 μg/μL) by a TRUEscript 1st Strand cDNA Synthesis Kit (Aidlab Biotechnologies, Beijing, China). 1 μL cDNA template and 0.5 μL primers were added to 12.5 μL 2×SYBR qPCR Mix(Aidlab Biotechnologies, Beijing, China). The amplification conditions for LPL gene and 18S rRNA were 94 °C for 3 min, denaturation at 95 °C for 10 s, annealing and extension at 65 °C for 60 s for a total of 40 cycles. Calculated mRNA levels were normalized to 18S RNA. The quantitative measurement of mRNA was performed on a Cobas Z480 Cycler (Roche, Switzerland).

The following primers were listed [33]:

LPL-F 5’-GCGTGATTGCAGAGAGAGGAC-3’

LPL-R 5’-TCAGGCAGAGTGAATGGGATG-3’;

18S rRNA-F 5’-GGAAGGGCACCACCAGGAGT-3’

18S rRNA-R 5’-TGCAGCCCCGGACATCTAAG-3’.

#### Analysis of LPL activity and mass

Transfected COS-1 cells (5×10^6^) were seeded into 100 mm dishes containing 10 mL DMEM medium supplemented with 10% fetal bovine serum and cultured for 2.5 days. After washing with PBS for 3 times, the COS-1 cells were treated with 0.5 mL cell medium combined with 1.6 μL heparin (20 units/mL) for 10 min at 37 °C. After repeating the procedure thrice, the 1.5 mL pooled medium was harvested and centrifuged at 4 °C for 10 min at 10,000g. The supernatant containing LPL released from cell surface was immediately frozen in liquid nitrogen for further measurement of LPL activity and mass. The remaining cells were washed with PBS and then solubilized in 431 μL cell lysis buffer (Beyotime Biotechnology, Shanghai, China) as well as 1.4 μL heparin (20 units/mL). The cell lysates were centrifuged at 4°C for 10 min at 10,000 g, and the supernatant was stored at -80 °C. LPL activity and mass were determined by a LPL activity assay kit (Roar biochemical) and a Human LPL ELISA kit (TSZ Biological Trade) respectively, as described above.

### Statistics

Statistical analysis was performed by the SPSS V23 software. TG levels were log-transformed before analysis, and all values were compared between HTG subjects and controls by Student's two-tailed t-test. One-way ANOVA was employed for comparison between different groups *in vitro* studies. P < 0.05 was deemed as statistically significant.

## SUPPLEMENTARY MATERIALS FIGURE



## Abbreviations

TG: triglyceride
TC: total cholesterol
HDL: high-density lipoprotein
LDL: low-density lipoprotein
VLDL: very-low-density lipoprotein
Lp(a): lipoprotein(a)
FFA: free fatty acid
HbA1c: hemoglobin A1c
LPL: lipoprotein lipase
APOC2: apolipoprotein C-II
APOA5: apolipoprotein A-V
GPIHBP1: glycosylphosphatidylinositol-anchored high density lipoprotein-binding protein 1
LMF1: lipase maturation factor 1
HL: hepatic lipase
ATCC: American type culture collection
DMEM: Dulbecco's modified Eagle's medium
PBS: phosphate buffer saline
SNPs: single nucleotide polymorphisms
APOE: apoprotein E
PCR: polymerase chain reaction
ELISA: enzyme linked immunosorbent assay
LPL-wt: LPL wild-type
rER: rough endoplasmic reticulum
FHTG: familial hypertriglyceridemia
PVDF: polyvinylidene difluoride
