# Supplementary material for: Severe hypertriglyceridemia due to two novel loss-of-function lipoprotein lipase gene mutations (C310R/E396V) in a Chinese family associated with recurrent acute pancreatitis
Source: Oncotarget. 2017 May 10;8(29):47741–54. doi: 10.18632/oncotarget.17762 (PMC5564601; doi:10.18632/oncotarget.17762)
Supplement: Supplementary file 1 [file oncotarget-08-47741-s001.pdf]

Severe hypertriglyceridemia due to two novel loss-of-function lipoprotein lipase gene mutations (C310R/E396V) in a Chinese family associated with recurrent acute pancreatitis

SUPPLEMENTARY FIGURE

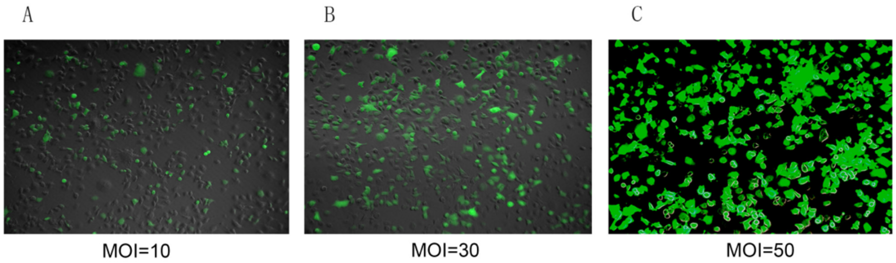

Supplementary Figure 1: Lentiviral transfection efficiency of different MOI shown by a fluorescence microscope(×10). It is suggested that MOI=50 exhibited the best transfection efficiency. MOI, multiplicity of infection.
